# Comparative genomic analyses of a virulent pseudorabies virus and a series of its in vitro passaged strains

**DOI:** 10.1186/s12985-018-1102-8

**Published:** 2018-12-29

**Authors:** Chao Ye, Jiqiang Wu, Wu Tong, Tongling Shan, Xuefei Cheng, Jingjing Xu, Chao Liang, Hao Zheng, Guoxin Li, Guangzhi Tong

**Affiliations:** 1 Shanghai Veterinary Research Institute, Chinese Academy of Agricultural Sciences, No. 518, Ziyue Road, Minhang District, Shanghai, 200241 China; 2 Jiangsu Co-innovation Center for Prevention and Control of Important Animal Infectious Diseases and Zoonoses, Yangzhou, 225009 Jiangsu China

**Keywords:** Pseudorabies virus, Genomic analyses, Passaging, Attenuation, Dynamic variation

## Abstract

**Background:**

Pseudorabies virus (PRV) of the family *Herpesviridae* is the causative agent of Aujeszky’s disease. Attenuation of PRV by serial passaging in vitro is a well-established method; however, the dynamic variations occurring on viral genome during this process have not been characterized.

**Methods:**

Genome sequencing and comparative genomic analyses of a virulent pseudorabies virus and a series of its plaque-purified strains via serial passaging in vitro were performed, and the properties in vitro and in vivo of which were further characterized.

**Results:**

Compared to the parental virus, replication in vitro was enhanced in the highly passaged F50, F91, and F120. In contrast, lethality in mice decreased gradually with passage number. Genome sequencing of F50, F91, and F120 showed deletion of a large fragment containing gE, which is likely related to their attenuation. In addition, single nucleotide variations were identified in many genes of F50, F91, and F120. In-frame and frameshift indels were also detected in specific genes of passaged strains. Particularly frameshift mutations were observed in highly passaged strains, resulting in a truncated but overexpressed pUL46.

**Conclusion:**

During attenuation of PRV by serial passaging in Vero cells, dynamic variation patterns including a large deletion, single nucleotide variations, small in-frame indels, and also frameshifts mutations successively emerged, contributing to evolution of the viral population and enabling the gradual attenuation of the virus. These data provide clues to better understand PRV attenuation during passaging.

**Electronic supplementary material:**

The online version of this article (10.1186/s12985-018-1102-8) contains supplementary material, which is available to authorized users.

## Introduction

Pseudorabies virus (PRV) of the family *Herpesviridae*, subfamily *Alphaherpesvirinae* [1], is the causative agent of Aujeszky’s disease, a major viral disease in pigs, the virus natural reservoir. It causes severe neurological disease and high mortality in newborn piglets, and reproductive failure in sows [2], resulting in significant economic losses to the pig industry worldwide. Besides pigs, PRV can infect numerous mammals causing neurological disease and acute death [3].

Effective vaccines have long been available for PRV [4]. Among the various vaccines used, the attenuated Bartha vaccine strain, a derivative of a virulent strain generated by extensive passage, has been the most commonly used [5]. Thus far, large-scale vaccination combined with the implementation of effective diagnostic tests to differentiate infected from vaccinated animals has allowed eradicating circulating PRVs from domestic pigs in many countries [6]. However, since 2011, a reemergence of pseudorabies has occurred in vaccinated pigs in China. Genomic analysis of PRV variants isolated from these outbreaks has shown that they are evolutionarily divergent from European-American strains [7], and lack of complete protection by the Bartha-K61 vaccine has been experimentally confirmed [8, 9]. This has prompted the need to fully comprehend virus attenuation for the development of new vaccine candidates.

Since the 1980s, a number of studies have been undertaken to identify the genome-wide mutations in Bartha and other vaccine strains, thereby elucidating the genetic basis of their attenuation. And a large deletion containing two nonessential glycoproteins (gI and gE) within the unique short (US) portion of the viral genome was identified and proven to contribute to virus attenuation [10–12]. Subsequent studies further showed that defects in other genes of live vaccine strain Bartha also contributed to its attenuated phenotype [13, 14]. More recently, Illumina high-throughput sequencing (HTS) was applied to determine the genomic diversity in this vaccine strain, resulting in the discovery of many previously unknown coding differences between Bartha and PRV virulent strains [15]. These studies have provided very important clues to understand attenuation and variation in PRV. However, if inclusion of viruses from the intermediate passages of the attenuation process in this type of studies might allow connecting more genetic variation information with specific phenotypic differences, thereby gaining clear insights into attenuation at the genetic level.

In our previous work we developed an attenuated PRV by serial passage of the variant JS-2012 in Vero cells at 40 °C for 120 generations [16]. Pathogenicity in piglets of the resulting strains F50, F91, and F120 (named according to passage number) showed that pathogenicity gradually declined as the number of passages increased, with JS-2012-F120 being avirulent in 2-week-old piglets [16]. But the relationship between virus attenuation and genetic variation during the process of serial passaging is still not clear.

To better understand the relationship between genetic variation and virus attenuation, in the present work we have further characterized these JS-2012 passaged PRV strains in vitro and in vivo. Compared to the parental virus, strains from passages 50 to 120 produced higher titers but relatively smaller plaques. Lethality of the viruses in a mouse model gradually decreased as the passage number increased. Genome-wide sequencing showed the presence of a large deletion, including genes US8, US9, and US2, in all the passaged strains analyzed. In addition, a variable number of single nucleotide variations were detected in many genes, mostly in the UL region of the genome. In addition, small in-frame and frameshift indels were identified in some genes. In particular, frameshift mutations were observed in genes UL16 and UL46, the latter producing a truncated but overexpressed pUL46, which might contribute to the enhanced virus replication in vitro and attenuation in animals. These data provide important clues to better understand attenuation and variation in PRV, and offer further insights into the evolution of the virus.

## Materials and methods

### Viruses and cells

PRV JS-2012 is a PRV variant isolated from a diseased newborn piglet. Strains JS-2012-F50, -F91, and -F120, named according to their respective passage, were purified from the corresponding virus stocks by three rounds of single-plaque cloning at 37 °C. Vero and PK-15 cells were grown in Dulbecco’s modified Eagle’s medium (DMEM) (Gibco, USA) supplemented with 10% fetal bovine serum (Gibco, USA).

### Infection of mice

A total of 50 SPF BALB/c mice from six- to eight-week-old were divided into 5 groups (10 mice per group). Groups 1 to 4 were inoculated intramuscularly with a 100-μL inoculum containing 10^4^ 50% tissue culture infective dose (TCID_50_) of JS-2012, F50, F91, and F120, respectively. Mice in group 5 were inoculated with 100 μL of DMEM and constituted the control group. Neurological symptoms and survival status of mice were observed every 12 h for the first 2 days after challenge, because during this period the mice generally did not show significant symptoms. During the remaining time of the monitoring period, mice gradually developed neurological symptoms and therefore were observed every 6 h for the rest 8 days in order to capture the changes of survival status of each mice. Meanwhile the neurological symptoms score level was determined for each mice in experimental groups. Specifically, mice with the absence of neurological symptoms were scored as 0, mild neurological symptoms such as unrest, excitation and occasional itching were scored as 1, and severe neurological symptoms (severe pruritus and self-mutilation) were scored as 2. Mice with a score of 2 were considered “dead”, and euthanized for animal welfare reasons [17]. All animal experiments were performed in accordance with the Guidelines for the Care and Use of Laboratory Animals of the institute and under the protocols approved by the Institutional Animal Care and Use Committee.

### Illumina library preparation and sequencing

The complete genome sequence of the JS-2012 strain (GenBank Accession no. KP257591) has been previously described [7]. The genomic DNA of the other strains was prepared as previously described [15]. Genomic library was prepared using Nextera XT DNA Sample Preparation Kit (Illumina, USA) and sequencing was performed on an Illumina Miseq platform, at the Shanghai Majorbio Bio-pharm Technology Co., Ltd. (Shanghai, China). The number of sequence reads generated for each strain is listed in Additional file 1: Table S1.

### PCR amplification and sanger sequencing

PCRs of several open reading frame (ORF) regions that could not be defined by HTS or of genes needing further validation were performed in 50 μL reactions containing 2 μL of template DNA (50 ng), 25 μL of 2 × GC buffer II (Takara), 0.5 μL of Ex Taq polymerase (Takara), 0.5 mM primers, deoxynucleoside triphosphates and distilled water. PCR products of the expected sizes were cloned into the pMD-18 T vector (Takara), and three randomly selected clones per PCR product were sequenced by Sanger sequencing.

### Annotation of genes and analysis of protein-coding sequences

The raw Illumina reads of F50, F91, and F120 were firstly deadaptered and merged into single longer reads by SeqPrep program, then the quality-controlled sequencing fragments were aligned with the reference genome (GenBank Accession no. KP257591) by BWA software and then assembled by geneious 8, respectively. PCR amplification and Sanger sequencing were used to determine the gaps. The genomes of F50, F91, and F120 were annotated by BLAST analysis of each viral ORF, with manual adjustment to the JS-2012 reference sequence. The annotated genomic sequences were deposited into the GenBank sequence database with accession numbers MG551316 (F50), MG551317 (F91), and MG589642 (F120). For single nucleotide variations analysis, the nucleotide sequence of each ORF of the three passaged strains and JS-2012 were aligned to identify the number and frequency of single nucleotide variations in each passaged strain using the algorithm Muscle (Codons) implemented in MEGA v.5.0 [18]. Meanwhile, all the variations identified in F50, F91, and F120 compared to JS-2012 at both the amino acid and nucleotide levels are summarized in Table 1 and Additional file 2: Table S2, respectively, and the in-frame and frameshift indels are described in Table 2.

**Table 1:** Amino acid variations identified in the F50, F91 and F120 passages of PRV JS-2012

Gene	Amino acid variations found in F50, F91, and F120 compared to JS-2012^a^		
	F50	F91	F120
UL6	P542S, P553S	P542S, P553S	P542S, P553S
UL8	S542A	S542A	S542A
UL10 (gM)	V256A	V256A	V256A
UL15	G169E, P669D	G169E, P669D	G169E, P669D
UL16	ND	317(+LPRH), S322P, 325–327(IPE > NKR), 329–330(IN>LK), 331–332(DY>△)	317(+LPRH), S322P, 325–327(IPE > NKR), 329–330(IN>LK), 331–332(DY>△)
UL17	G237D, A241S, T249A, P253△, A255P, D258A, 259(+GGG), N263D, P273L, P374L	G237D, A241S, T249A, P253△, A255P, D258A, 259(+GGG), N263D, P273L, P374L	G237D, A241S, T249A, P253△, A255P, D258A, 259(+GGG), N263D, P273L, P374L
UL18 (VP23)	H47Y, P60A, H69Q, S79G, A142V, T270 M	H47D, P60A, S79G, A142G, T270 M	H47D, P60A, S79G, A142G, T270 M
UL19 (VP5)	I178M, I1315T	I178M	I178M
UL22 (gH)	P433L, A618V	P433L, A618V	P433L, A618V
UL25	L23P	L23P	L23P
UL26 (VP24)	M124 T, I125L, R131L, S132C, Q136R, S137R, R139G, L143 V, T146A, V153A, Q161R	M124 T, I125L, R131L, S132C, Q136R, S137 V, R139G, L143 V, T146A, V153A, Q161R, A455△	M124 T, I125L, R131L, S132C, Q136R, S137R, R139G, L143 V, T146A, V153A, Q161R
UL26.5	ND	A216△	ND
UL28 (ICP18.5)	A413P, D414E, D425G, 425(+GA), V430G, D432G, A522V	A413P, D414E, D425G, V430G, D432G, A522V	A413P, D414E, D425G, 425(+GA), V430G, D432G, A522V
UL33	P39A	P39A	P39A
UL34	A177V, T178S	A177V, T178S	A177V, T178S
UL36 (VP1/2)	T2832A	T2832A	T2832A
UL37	E240D, F629 L, G762R	E240D, F629 L, G762R	E240D, F629 L, G762R
UL38 (VP19c)	A218V	A218V	A218V
UL40 (RR2)	A176T	A176T	A176T
UL44 (gC)	R107H	G90D, R107H	G90D, R107H
UL46 (VP11/12)	ND	ND	599–626(PLTRHGSMRTSFRRGVRAAQRFVRRRLS>△), 629–631(SAE > TTT), A633P, 635–674(RASGDSASAAAPAAASARGETDHVYQHPRPRTRADDGLYQ>△), Q675G, 678–695(PVIDLTGHRASRRKSWRV>△)
UL48 (VP16)	R39Q,P89A	R39Q,P89A	R39Q,P89A
UL49 (VP22)	R168H, N198D	R168H	R168H
UL49.5 (gN)	T87A	T87A	T87A
UL50 (dUTPase)	S209A	S209A	G191R,S209A
UL53 (gK)	P164L, P171L	P164L, P171L	P164L, P171L
UL54 (ICP27)	W20R, C48R, S156F,Q182R	W20R, C48R, S156F,Q182R	W20R, C48R,S156F,Q182R
US8 (gE), US9, US2	Deletion	Deletion	Deletion
IE180 (ICP4)	P468S, G1385R	S187 L, P468S, G1385R	L76P, P468S, G1385R

*ND* referred to no difference

^a^Single amino acid residues changes were recored in the following format, including the JS-2012 reference strain amino acid, its position, and the amino acid residue found in the passaged strains. Insertions were indicated by the amino acid position in JS-2012 followed by “+” and the new amino acid in passaged strains. Deletions were indicated by the symbol △. Sequential changes are shown with the JS-2012 amino acid positions first, followed by the relevant JS-2012 amino acid residues, then with “>”, and finally the alternative amino aicd residues of passaged strains

**Table 2:**
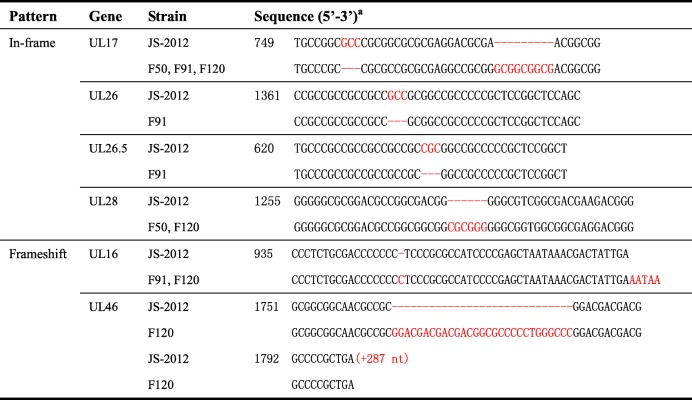
Indels identified in passaged strains compared to the parental virus (JS-2012)

^a^Numbers show the initial position of the sequence relative to the ATG of the corresponding ORF in JS-2012. Nucleotide insertions are shown in red and deletions are represented with hyphens. The “+ 287 nt” indicates 287 additional nucleotides at the 3′ end of the UL46 ORF of JS-2012 compared to that of F120

### 3′ rapid amplification of cDNA ends (RACE)

The total RNA of PK-15 cells infected with the indicated virus was extracted using the RNeasy Mini Kit (Qiagen) and treated with RNase-free DNase I (Ambion) followed by reverse transcription with the primer Q_T_ 5’-CCAGTGAGCAGAGTGACGAGGACTCGA(T16)-3′. The PCR reactions for obtaining the mRNA 3′ ends of UL16 and UL46 were performed with the resulting cDNA product and the following primers: UL16 F597/Q_0_ (5’-CGAGTGCCGCGTGGACCAC-3′ and 5’-CCAGTGAGCAGAGTGACG-3′) and UL46 F453/Q_0_ (5’-GCACCCGTTCAAGCACAAG-3′ and 5’-CCAGTGAGCAGAGTGACG-3′). UL16 F597 and UL46 F453 are the oligos annealing with the UL16 and UL46 genes, while Q_0_ is the anchor primer of Q_T_. The PCR fragments were sub-cloned into the TA cloning vector pMD-18 T (Dalian, China) and subjected to DNA sequencing.

### Western blot analysis

At 24 h post infection, cells were collected into ice-cold PBS, centrifuged, and lysed with RIPA buffer for 30 min followed by centrifugation at 4 °C for 3 min. The supernatants were collected, boiled for 10 min, separated by SDS-PAGE on a 10% polyacrylamide gel, and transferred to a nitrocellulose membrane using a Bio-Rad semi-dry transfer cell. The membranes were blocked using 5% non-fat milk in TBS-T buffer, incubated with mice polyclonal antibodies for VP5 (UL19) (1:1000), UL16 (1:500), and UL46 (1:500), and mice monoclonal antibodies for gE (1:1000) and β-actin (1,6000), diluted in 2% non-fat milk in TBS-T buffer, followed by incubation with goat horseradish peroxidase-conjugated secondary antibodies diluted in TBS-T buffer. Protein band intensities were measured using the ImageJ (NIH) Gel Analyzer module.

### ORF analysis of UL46 and UL16 containing frameshift mutations

The ORFs of UL46 and UL16 containing frameshift mutations were analyzed using ORF Finder at the National Center for Biotechnology Information (https://www.ncbi.nlm.nih.gov/orffinder/) with the default setup, and EditSeq (DNASTAR) was used to manually calculate the molecular weights of the corresponding proteins of each virus strain.

## Results

### Serial passaging at high temperature generated attenuated strains with high titer

**Fig. 1:**
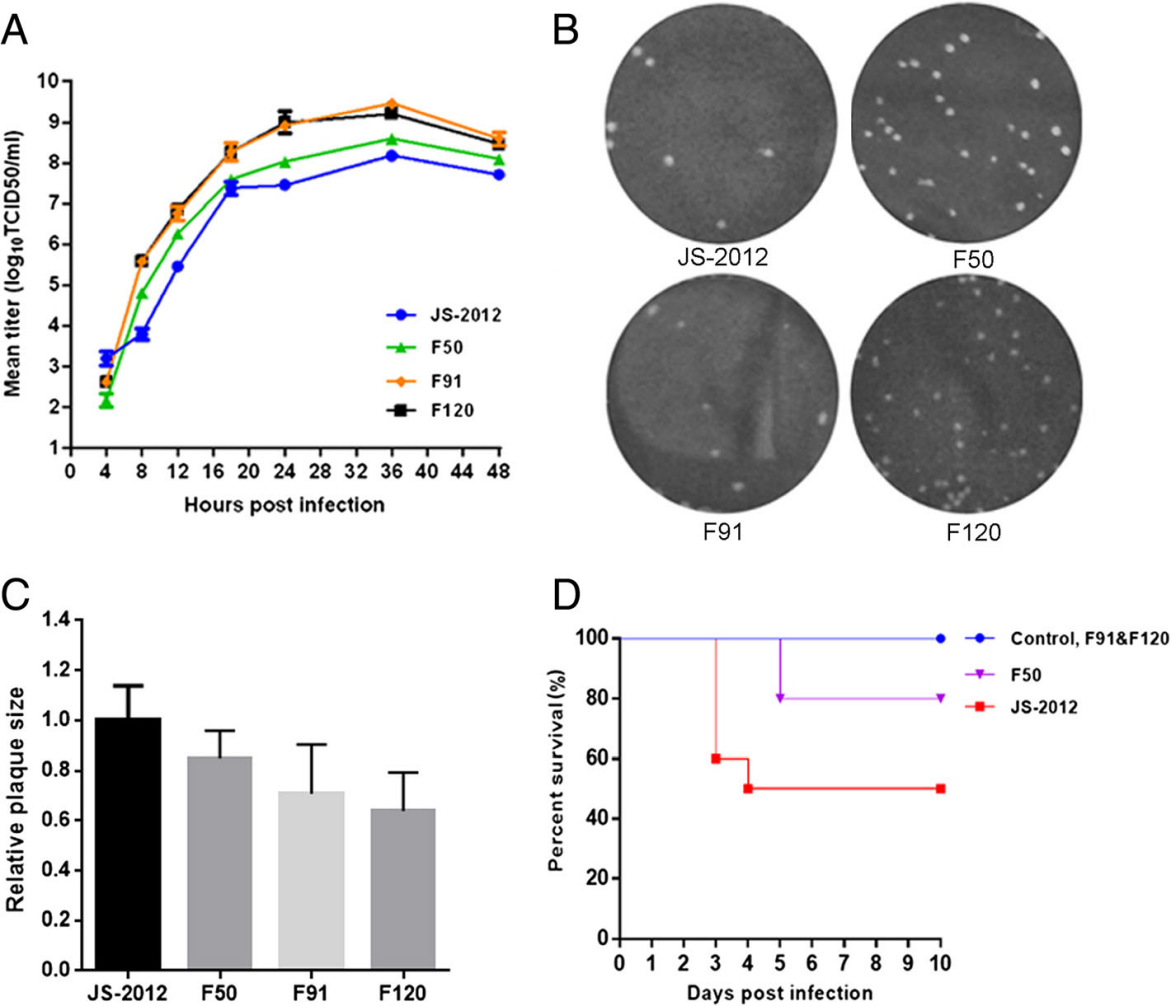
In vitro and in vivo characteristics of the JS-2012 and its passaged strains. **a** One-step growth curves. PK-15 cells were infected at an MOI of 1 with each virus. Cell culture supernatants were harvested at 4, 8, 12, 18, 24, 36, and 48 h post infection. Virus titers at each time point were determined by the TCID_50_ assay in Vero cells. The data represented means ± SD for 2 independent experiments per data point. **b** Plaques of JS-2012 and the serial passaged strains generated in infected PK-15 cells cultured at 37 °C for 4 days. **c** Relative plaque diameters of each virus were calculated and compared to those of PRV JS-2012. Meanwhile the average plaque diameter of PRV JS-2012 were set as 1. **d** Survival percentages of mice inoculated with 10^4^ TCID_50_ of each virus per mouse. In JS-2012-infected group, a total of 2 mice were heavily infected and dead at 3 days post inoculation, and another 3 mice with severe neurological symptoms (self-mutilation) were euthanized respectively for animal welfare reasons; in F50-infected group, 2 of 10 mice exhibited severe pruritus and self-mutilation symptoms and were euthanized for animal welfare reasons at 5 days post inoculation; The remaining 43 mice in control group and JS-2012, F50, F91, F120-infected groups were survived and then euthanized till the end of the experiment

To further characterize the replication properties of the passaged strains, strains from various points of the serial passage were selected and purified for evaluation. One-step growth kinetics in PK-15 cells showed that the PRV strains from higher passages (F50 to F120) exhibited a higher replication rate within the first 12 h post infection and higher viral titers at the later time points analyzed (Fig. 1a). In contrast, the plaques of F50, F91 and F120 grew a little smaller than that of JS-2012 gradually (Fig. 1b-c). Furthermore, in a mouse model, lethality of the passaged strains gradually decreased as passaging increased, with F50 still retaining a certain degree of lethality, while F91 and F120 showed no lethality in mice (Fig. 1d).

### Single nucleotide variations occurring during PRV passage

**Fig. 2:**
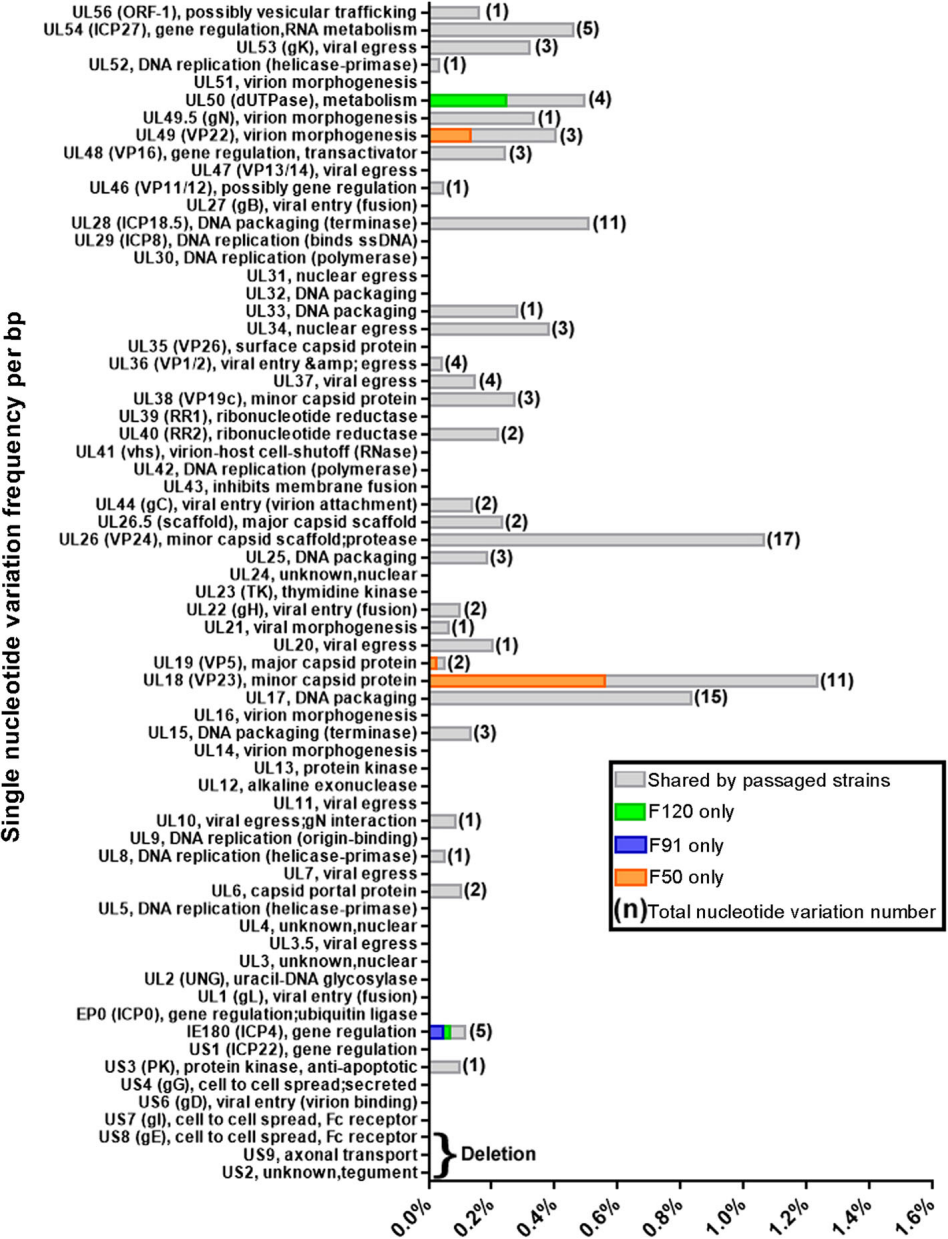
Summary of single nucleotide variations identified in F50, F91 and F120, versus the parental strain JS-2012. Gene names and functions were listed on the left. Single nucleotide variations identified in each virus were categorized as being unique to F50 (orange), unique to F91 (blue), unique to F120 (green) or shared (observed in two or three of the passaged strains; gray). And the three genes deleted in passaged strains were bracketed at the bottom

To better understand the genetic variation of the observed phenotypes, the whole genome of strains F50, F91 and F120 were sequenced using a combination of Illumina and Sanger sequencing. The sequences of all the ORFs were compared to define the inter-strain diversity in PRV JS-2012 and its passaged strains. Variation analysis showed that many genes contained single nucleotide variations. Specifically, 119 variable sites were identified among the three passaged strains and JS-2012 (Fig. 2). Single nucleotide variations occurred mainly in the UL region of the virus genome, in particular in UL28, UL26, UL18, and UL17, each of which contained more than 10 single nucleotide variation sites (Fig. 2). Consequently, the single nucleotide variations frequency (number of single nucleotide variations/ORF length) of UL28, UL26, UL18, and UL17 were very high at 0.5, 1.0, 1.2, and 0.8%, respectively (Fig. 2). No more than 5 variation sites were found in any other gene and, therefore, their incidence frequency were below 0.5% (Fig. 2).

### Large deletions and small indels are involved in the evolution of PRV during serial passages

One of the most notable variations identified was a large deletion covering the region of US8 (gE), US9, and US2 (Table 1). The US8 (gE) deletion is also present in the genome of Bartha and other vaccine strains attenuated by extensive in vitro passage. In addition, we found small indels in the genomes of passaged strains. In-frame indels were present in the ORFs UL17, UL26, UL26.5, and UL28, and some, but not all, were related to number variation of short sequence repeats. For instance, a 3-nt deletion in UL26 of strain F91 was apparently caused by deletion of one repeat of the short sequence repeat “GCC” (Table 2).

**Fig. 3:**
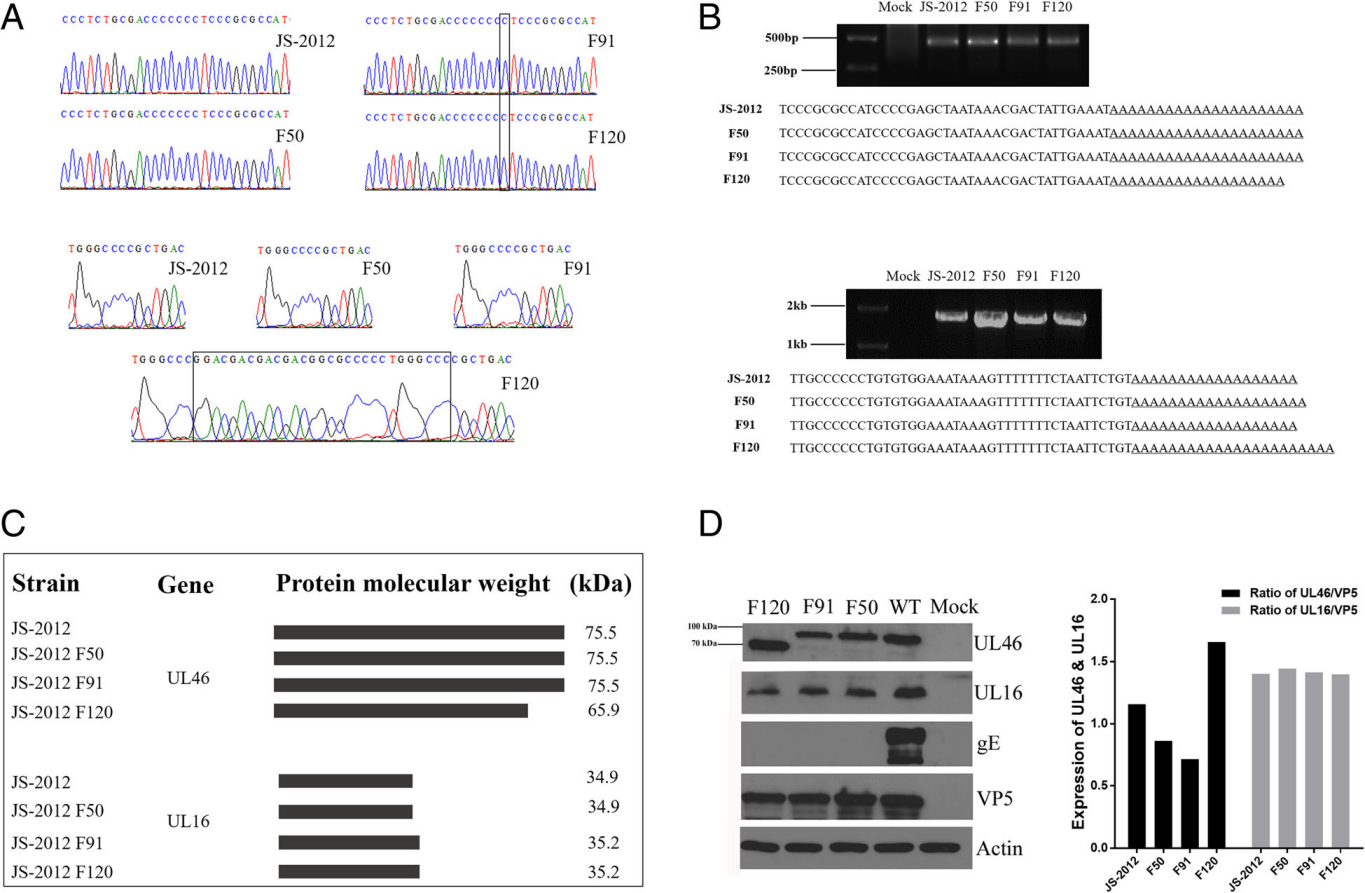
Analysis of the frameshifts mutations occurred in the UL46 and UL16 genes during serial passage. **a** Sequencing data of the 3’ RACE of UL16 (top) and UL46 (bottom) of each strain. The positions of the frameshift mutations are marked with black rectangles. **b** Transcription termination analysis of UL16 (top) and UL46 (bottom) in each strain. The results of the 3’ RACE PCR amplification and sequencing data with the transcription termination site and the poly(A) sequences underlined are shown. **c** The length of the ORFs of each strain were predicted with ORF Finder and the corresponding protein molecular weights were then calculated using the EditSeq module implemented in DNASTAR. **d** Western blot analysis of protein levels inter-strain variation of UL46 and UL16. Cell lysates were hybridized with anti-gE antibodies to confirm deletion of gE in the passaged strains. The capsid protein VP5 is shown for comparison and as a loading control. Actin was also detected as a loading control. The ratios of UL46 or UL16 versus VP5 in each sample were calculated using the ImageJ Gel Analyzer module

Frameshift mutations were also identified. Sequencing at both the DNA and mRNA levels confirmed the presence of frameshift mutations in the UL16 and UL46 genes of some passaged strains. Specifically, the mutation (an insertion of a cytosine) in UL16 of F91 and F120 occurred within a homopolymer (CCCCCCC) nucleotide stretch, whereas the frameshift mutation in UL46 of F120 was caused by the insertion of a 29-nt short sequence repeat (GGACGACGACGACGGCGCCCCCTGGGCCC) (Fig. 3a). Specific 3’ RACE of these genes further revealed that the two frameshift mutations had no effect on transcription termination of the two genes; that is, the UL16 and UL46 ORFs of all the strains studied were transcribed and terminated at the same position (Fig. 3b). However, bioinformatic analysis of these ORFs indicated that the mutations resulted in a premature termination codon in the UL46 of F120 (Fig. 3c). Indeed, the predicted 65.9-kDa truncated UL46 product was detected by Western blot analysis of the cell extracts of F120-infected cells (Fig. 3d). On the other hand, as predicted by ORF Finder the frameshift mutation in UL16 gene resulted in a delayed termination codon in that of F91 and F120 (Fig. 3c). However, this mutation resulted in only a 0.3-kDa change in protein size, which cannot be detected by western blot analysis (Fig. 3d). In addition, the frameshift mutation in UL46 were associated with relatively higher expression levels of the corresponding product in F120 compared to the other strains, while the mutation in UL16 had no significant effect on the expression of its protein product in F91 and F120 (Fig. 3d).

## Discussion

Viruses can acquire mutations as they replicate in cell culture, while only mutations favorable to viral replication in vitro are rapidly selected and enriched in the population. This is particularly significant during extensive passage in culture, resulting in viruses with novel acquired genetic alleles and evolved phenotypes that usually less pathogenic in the natural host but better adapted for replication in cells [19, 20].

In our previous study, the PRV JS-2012 strain was serially passaged at high temperature for 120 generations. As the passage number increased, viruses showed a progressive attenuation in the piglet infection model, up to the point of completely losing its ability to cause death in piglets by passage 120 [16]. To characterize the evolution and variation of the viral population during passaging, in this study, several representative strains at various steps of the serial passage were characterized in vitro and in vivo, and the genome of F50, F91, and F120 was sequenced and further analyzed. The growth curves and plaque assays showed that, during passaging the viruses were better adapted for replication and release in the cell line, but seem that the ability to spread were a little weakened (Fig. 1). In addition, the lethality of the passaged strains in mice decreased progressively as the passage number increased, which is consistent with our previous results in piglets (Fig. 1). Therefore, during in vitro passaging the virus gained better replication capacity in cells while becoming largely attenuated in animals, probably as a result of genetic variation and rapid evolution of the virus.

To gain insights into the evolution of JS-2012 during continuous in vitro passage, three representative strains (F50, F91, and F120) were sequenced and analyzed. Notably, a deletion of large DNA fragment including US8 (gE), US9, and US2 was identified in the genomes of all three strains. Absence of gE expression in the three passaged strains was further confirmed by Western blot analysis (Fig. 3d). Several studies have shown that deletion of gE from the PRV genome causes attenuation of the virus [21, 22]. Therefore, attenuation of JS-2012 during passaging is likely to be related to deletion of the fragment containing gE. The presence of large deletions or deletion of some genes is very common in other herpesviruses and in large DNA viruses extensively passaged in culture [23], suggesting that this might be a unique feature of large DNA viruses evolving in cell culture. Besides the large deletion, single nucleotide variation was a dominant variation pattern in many genes, particularly in the UL region of the virus genome. In addition, small in-frame indels identified in genes UL17, UL26, UL26.5, and UL28 contributed to evolution of the virus. Some of these indels seemed to have occurred by contraction of specific short sequence repeats, most likely through recombination or polymerase slippage mechanisms [15]. But the remaining indels were not related to short sequence repeats, suggesting other mechanisms might be involved in the generation of indels during the evolution of PRV.

Previously frameshift mutations have been observed in the genome of PRV by serial passaging in rabbit kidney cells [24], and also in the TK gene of Acyclovir-resistant HSV-1 strains and the RL5A, RL13, UL131A and UL130 genes of human cytomegalovirus [25, 26]. And it was confirmed that the frameshift mutations might be relevant for the adaptation of PRV in cell culture [24]. Here frameshift mutations also occurred during serial passaging of PRV JS-2012 in Vero cells. The presence of the frameshift mutations was confirmed at both the DNA and mRNA levels for the UL16 and UL46 genes in the corresponding passaged strains. An insertion of a cytosine within one homopolymer nucleotide stretch (CCCCCCC) generated a frameshift in the UL16 gene of F91 and F120, and the frameshift in the UL46 gene of F120 was generated by an insertion of a 29-nt short sequence repeat (GGACGACGACGACGGCGCCCCCTGGGCCC). Therefore, both mutations were related to alterations in short sequence repeats. Specific 3’ RACE further revealed that the two frameshift mutations had no effect on transcription termination of any of the two genes, suggesting that the transcriptional stop signal of the viral gene had been conserved. Further analysis suggested that the mutations resulted in a premature termination codon in the UL46 of F120, and a delayed termination codon in the UL16 of F91 and F120 (Fig. 3c). Accordingly, the predicted 65.9-kDa truncated UL46 product was detected by Western blot analysis in F120-infected cell extracts (Fig. 3d). Moreover, the truncated UL46 product expressed by F120 was expressed at higher level than the full-length protein produced by the lower passage viruses. The effect of these changes on virus growth and attenuation deserves further investigation.

In conclusion, extensive passaging of PRV in vitro can result in a great deal of variation, dramatically changing the biological characteristics of the virus. In this study, we showed that during attenuation of PRV by serial passaging in Vero cells, dynamic variation patterns including a large deletion, single nucleotide variations, small in-frame indels, and frameshifts mutations successively emerged contributing to evolution of the viral population and enabling the gradual attenuation of the virus. In particular, the frameshift mutation in UL46 affected the size and expression level of the corresponding proteins and could have a potentially important effect on the virus characteristics. All these data provide important clues to better understand attenuation and variation in PRV, and further offer insights into the evolution of the virus.

## Conclusions

During attenuation of PRV by serial passaging in Vero cells, dynamic variation patterns including a large deletion, single nucleotide variations, small in-frame indels, and frameshifts mutations successively emerged contributing to evolution of the viral population and enabling the gradual attenuation of the virus. In particular, the frameshift mutation in UL46 affected the size and expression level of the corresponding proteins and could have a potentially important effect on the virus characteristics. All these data provide important clues to better understand attenuation and variation in PRV, and further offer insights into the evolution of the virus.

## Additional files

Additional file 1: **Table S1.** The raw data of illumina sequencing. (DOCX 13 kb)

Additional file 2: **Table S2.** Nucleotide variation in ORFs of F50, F91, F120 compared to JS-2012. (DOCX 16 kb)

## Abbreviations

DMEM: Dulbecco’s modified Eagle’s medium
HTS: High-throughput sequencing
ORF: Open reading frame
PRV: Pseudorabies virus
RACE: Rapid amplification of cDNA ends
TCID_50_: 50% tissue culture infective dose
