# Supplementary material for: Comparative genomic analyses of a virulent pseudorabies virus and a series of its in vitro passaged strains
Source: Virol J. 2018 Dec 29;15:195. doi: 10.1186/s12985-018-1102-8 (PMC6310976; doi:10.1186/s12985-018-1102-8)
Supplement: Supplementary file 1 — Table S1. The raw data of illumina sequencing. (DOCX 13 kb) [file 12985_2018_1102_MOESM1_ESM.docx]

Table S1 The raw data of illumina sequencing

| Strain | Read length | Raw data (Mb) | Raw reads number | Q20 (%) | Average coverage | Clean data (Mb) | Clean reads number |
| --- | --- | --- | --- | --- | --- | --- | --- |
| F50 | 301PE | 599.42 | 1,991,444 | 69.63% | 4125X | 386.79 | 1941461 |
| F91 | 301PE | 803.02 | 2,667,828 | 70.58% | 5526X | 523.35 | 2587483 |
| F120 | 301PE | 712.99 | 2,368,736 | 66.73% | 4907X | 437.91 | 2268972 |
