# Supplementary material for: Comparative genomic analyses of a virulent pseudorabies virus and a series of its in vitro passaged strains
Source: Virol J. 2018 Dec 29;15:195. doi: 10.1186/s12985-018-1102-8 (PMC6310976; doi:10.1186/s12985-018-1102-8)
Supplement: Supplementary file 2 — Table S2. Nucleotide variation in ORFs of F50, F91, F120 compared to JS-2012. (DOCX 16 kb) [file 12985_2018_1102_MOESM2_ESM.docx]

**Table S2 Nucleotide variation in ORFs of F50, F91, F120 compared to JS-2012**

| Gene | Nucleotides identified in F50, F91&F120, which differ from JS-2012^a^ |
| --- | --- |
| UL6 | C1624T, C1657T |
| UL8 | T1624G |
| UL10 (gM) | T767C |
| UL15 | G506A, C2005G,C2006A |
| UL16 | 951(+CTCCCGCGCCAT), T952C, C957A, C960T, 962-969(TCCCCGAG> ATAAACGA), A973T, A975G, 978-979(CG>AT), C981A, 982-987(TATTGA>△) |
| UL17 | C708G, G710A, A711C, A714C, G721T, G723C, G741C, A745G, G753C, 755-757(CGC>△), G763C, A773C, 777(+GGCGGCGGC), A778G, C809T, C1112T |
| UL18 (VP23) | C27G, G30T, A108G, G111T, C139T, C178G, C207G, C228T, A235G, C425T, C809T |
| UL19 (VP5) | A534G,T3944C |
| UL20 | C396T |
| UL21 | G924A |
| UL22 (gH) | C1298T, C1853T |
| UL25 | C66G, T68C, T69C |
| UL26 (VP24) | T371C, A373C, T390G, G392T, G393C, A394T, A405G, A407G, A409C, C415G, C427G, A436G, G444C, T458C, A482G, A922C, C948G, 1361-1363(CCG>△) |
| UL26.5 | A184C, C210G, 639-641(CCG>△) |
| UL28 (ICP18.5) | G1237C, C1242G, A1274G, 1275(+GGCGCG), T1283G, C1284T, A1289G, A1293G, G1356C, A1536G, C1559T, C1560G |
| UL33 | C115G |
| UL34 | T468C, C530T, A532T |
| UL36 (VP1/2) | A1068C, C2577G, G3330C, A8494G |
| UL37 | G720C, T1885C, G2284C, C2346G |
| UL38 (VP19c) | C642G, C653T, T666C |
| UL40 (RR2) | A330G, G526A |
| UL44 (gC) | G269A, G320A |
| UL46 (VP11/12) | A1671G, 1784(+CCCCTGGGCCCGGACGACGACGACGGCGC), 1802-2088(CGCGCCACGGGAGCATGCGCACCAGCTTCCGGCGCGGGGTCCGCGCGGCCCAGCGCTTCGTGCGCCGCCGGCTCTCGCGCACGAGCGCCGAGGCGGCCCCGCGGGCCTCCGGCGACTCAGCCTCCGCGGCGGCCCCCGCCGCCGCCTCCGCCCGCGGCGAGACCGACCACGTGTACCAGCACCCCCGCCCGCGGACCCGCGCGGACGACGGCCTGTACCAGCAACCCCGACCCGTCATCGACCTCACCGGCCACCGCGCGTCGCGCCGCAAGAGCTGGCGCGTGTGA>△) |
| UL48 (VP16) | T43C, G116A, C265G |
| UL49 (VP22) | C78T, G503A, A592G |
| UL49.5 (gN) | A259G |
| UL50 (dUTPase) | A459G, G571C, C573G, T625G |
| UL52 | A1236G |
| UL53 (gK) | T363C, C491T, C512T |
| UL54 (ICP27) | T58C, T142C, C467T, A545G, A717G |
| UL56 | G552A |
| US8 (gE), US9, US2 | Deletion region of F50, F91 and F120 |
| US3 (PK) | C858T |
| IE180 (ICP4) | T227C, T559C, C560T, C1402T, G4153C |

^a^Single nucleotide changes were recored in the following format, including the JS-2012 reference strain nucleotide, its position, and the nucleotide found in the passaged strains. Insertions were indicated by the nucleotide position in JS-2012 followed by ‘‘+’’ and the new nucleotide in passaged strains. Deletions were indicated by the symbol △. Sequential changes are shown with the JS-2012 nucleotide positions first, followed by the relevant JS-2012 nucleotides, then with “>”, and finally the alternative nucleotides of passaged strains.
